# Editorial

**Published:** 1839-05-25

**Authors:** 


### THE MEDICAL EXAMINER.

PHILADELPHIA, MAY 25, 1839.

We learn from the Boston Medical and Surgical Journal, that Professor Gross of the Cincinnati Medical College, is about to publish at Boston, a new work on Pathological Anatomy. Dr. Gross has been assiduously engaged upon this subject for some years. We understand that his opportunities have been good; and from his ability as an investigator, we doubt not that the work will supply a deficiency now existing.

Two good works of pathological anatomy have been in the hands of the profession in this country; one, the reprint of Townsend's Translation of Andral's work, and the other written by Professor Horner, of the University of Pennsylvania. The progress which pathological anatomy has made, is so great, that a third work is wanted, and will undoubtedly be of great benefit to the practitioners of this country who have few opportunities of examining the pathological appearances of disease.

Dr. Gross's work has numerous illustrations, which are said to be well executed. These will add greatly to its usefulness. The large works of Carswell and Cruveilhier are by far too expensive for the immense majority of physicians; and without correct representations of diseased structure, it is extremely difficult to appreciate the nature of most morbid changes in the character of organs.

